# Play, Playfulness, and Self-Efficacy: Parental Experiences with Children on the Autism Spectrum

**DOI:** 10.1155/2018/4636780

**Published:** 2018-10-01

**Authors:** Rosa Román-Oyola, Verónica Figueroa-Feliciano, Yoliannie Torres-Martínez, Jorge Torres-Vélez, Keyshla Encarnación-Pizarro, Samariz Fragoso-Pagán, Luis Torres-Colón

**Affiliations:** University of Puerto Rico, Medical Sciences Campus, School of Health Professions, Occupational Therapy Program, PO Box 365067, San Juan, PR 00936-5067, USA

## Abstract

**Background:**

Play serves as an essential medium for parent-child interaction; however, engaging children with ASD through play can be a challenge for parents.

**Purpose:**

The purpose of this phenomenological study was to explore the perspectives of parents with children on the autism spectrum regarding play experiences and self-efficacy during play encounters.

**Method:**

Semistructured interviews were administered to 8 parents of children 3–7 years of age with ASD. The analysis was guided by the constant comparison method.

**Findings:**

Parental narratives denoted playful experiences reflecting components of Skard and Bundy's model of playfulness. The facilitation of framing and suspension of reality were generally more challenging than facilitating intrinsic motivation and internal control. Participants associated self-efficacy during play with their perceived ability to interact with their child and with positive emotions experienced during play. Fathers generally derived a greater sense of self-efficacy from play encounters than mothers, and this was explained by differences in fathers' and mothers' motivations for playing. Mothers were motivated to play for outcome-oriented reasons (e.g., promote the child's progress) whereas fathers' motivations depicted greater emotional emphasis, reflecting a better match between motivation and perceived indicators of efficacy during play.

**Conclusion:**

The results suggest that a good match between motivation for playing and perceived indicators of efficacy during play is important for a parental sense of self-efficacy. Occupational therapists should utilize coaching strategies to increase parents' understanding of play and playfulness and how they can affect a sense of parental self-efficacy.

## 1. Introduction

Playing has an important and primary role in childhood. As a child's primary occupation [1], play is as natural as breathing [2]. Play involves a child's active participation in an activity or time spent with peers and involves agency and the ownership of ideas [3]. During play, children learn important motor, cognitive, and social skills as well as creativity and self-confidence, which are skills that are required throughout life [1, 4, 5].

Children approach play in different ways based on different motivations and dispositions. These motivations and dispositions form the subject's attitude during play, also known as playfulness. Skard and Bundy [6] proposed a model of playfulness that identifies four primary characteristics of play. The first characteristic is *framing*, which refers to behaviors used during play that identify the nature of the activity as play. Players give framing cues to others, and good players must be able to give and read cues. The second characteristic of play is that it is *intrinsically motivated*. This means that players engage in the play activity because they enjoy the activity and experience benefits as a result. The third characteristic of play is *internal control*; this characteristic allows players to decide what they want to play, who they want to play with, and how and when the play should end. The fourth and final characteristic is *freedom to suspend reality*, which determines how closely a play transaction resembles the objective reality. Different strategies are employed to facilitate the suspension of reality. A player can pretend that he is someone else or that an object is something other than what it really is (e.g., pretends to be a chef; pretends that a box is an airplane). Players can also suspend reality by teasing or telling jokes [6, 7].

According to Skard and Bundy's model [6], a child's level of playfulness is determined by the summation of all the aforementioned characteristics. Evidence indicates that playfulness can differ among children with various developmental challenges. Playfulness in children with autism spectrum disorders (ASD) is affected by behaviors inherently related to their condition; children with ASD tend to be less playful than their typically developing counterparts [8, 9]. Behavioral challenges for play in children with ASD include fixed interests, lack of flexibility, impaired social skills, engagement in ritual repetitive behaviors, low levels of pretend play, anger or frustration, hyper- or hyposensitivities, and difficulty understanding nonverbal cues [10, 11].

Evidence suggests that the inability of some children with ASD to fully engage in play has negative effects on parents' as well as the child's well-being and sense of self-efficacy [12, 13]. Bandura [14] defined self-efficacy as an individual's belief in their ability to achieve a goal or outcome. Parental self-efficacy refers to the confidence and expectations of a parent regarding their ability to perform the parental role competently and effectively [15]. Compared to parents of typically developing children, parents of children with ASD experience higher levels of stress, depression, and hopelessness [16–18]. Participation in and enjoyment of play have important benefits for both parents and children, especially in families of children with ASD. Adult playfulness is a significant predictor of emotional parental self-efficacy among parents raising children with ASD, more so than the degree of sensory processing impairment inherently related to the child's diagnosis [19].

Playfulness is a relationship-based phenomenon and an important factor for the development of social relationships between children and their main caregivers [2, 20, 21]. Given social and communication difficulties in children with ASD, their parents may feel incompetent in their ability to establish an emotional connection with them. Additionally, the quality of involvement of the parents is an important variable for the ability of children with ASD to participate in play [22–24] and can also benefit the parents. Parents of young children with ASD that actively participate in coaching processes or guided educational treatments based on the use of strategies to maximize engagement opportunities with their child exhibit increased competence and higher levels of effect [13, 25].

Available evidence supports a potential relationship between play, playfulness, and parental self-efficacy, especially in families raising young children on the autism spectrum. Yet few studies have examined the experiential components underlying this relationship. The purpose of this phenomenological study was to explore the perspectives of parents with children on the autism spectrum regarding play experiences and self-efficacy during play encounters. The study addressed two research questions: (1) How do parents' narratives about play encounters reflect the elements of a playful experience for their child (based on the model of Skard and Bundy)? (2) How efficient do fathers and mothers feel when playing with their child with ASD?

## 2. Methods

### 2.1. Study Design

This study was guided by a phenomenological design with an interpretative approach. Interpretative approaches within phenomenology allow ample examination of relationships and the significance of knowledge and contexts [26]. Interpretative approaches are philosophically associated with social constructivism, which emphasizes the subjective meaning of an individual's experiences [27]. Phenomenology studies these subjective meanings to explicate the structure or essence of the lived experience in search of the unity of meanings [28]. Our research involved a detailed examination of the significance of participant play experiences with their children with ASD, which we defined as the phenomenon under study.

### 2.2. Participants

All study procedures were reviewed and approved by the Institutional Review Board of the University of Puerto Rico, Medical Sciences Campus. Four couples of parents of children with ASD from different parts of Puerto Rico consented to participate in semistructured in-depth interviews. The inclusion criteria were as follows: (a) availability of both parents for participation, (b) parents ≥ 21 years of age, and (c) children 3–7 years of age with a diagnosis of ASD. Age range selections were made based on previous investigations of the importance of reciprocal interactions between parents and children [29].  Table 1 describes some characteristics of participant parental couples.

### 2.3. Recruitment

Participants were recruited through direct contact or by referral from occupational therapists of children with ASD. In cases of recruitment by referral, occupational therapists were asked to make the first contact with parents regardless of whether the children were currently receiving therapy. After parents communicated interest in the study, we personally delivered a participation package including two consent forms (one for each parent), a sociodemographic information data form and an instruction sheet. Interviews with participants were scheduled at the participants' convenience. To minimize the possibility of bias associated with social desirability and interviewers' gender in participant narratives [30, 31], fathers were interviewed by male researchers and mothers were interviewed by female researchers.

### 2.4. Data Collection and Analysis

Data were collected via in-depth semistructured interviews. Open-ended questions were employed to allow participants to interpret the meaning of the question and respond based on their personal feelings and perceptions. Probe questions were included as part of the protocol and used to enrich the discussion as needed. Examples are as follows:

Main question: how do you feel when playing with your son/daughter?

Possible probes: how satisfied do you feel with the way you play? Do you think that you interact effectively while playing? What makes you think that way? Is there anything you would change about those playing moments?

Interviewing researchers were trained in qualitative methods and use of the interview protocol. Prior to the study data collection, all researchers performed practice interviews with volunteer mothers and fathers of typically developing children using the study protocol. Mothers and fathers were interviewed separately. The length of each study interview varied by participant in accordance with their comments and experiences but generally lasted approximately 45 minutes to an hour. Interviews were audio-taped with the participants' consent for later transcription in Spanish. All identifying information was removed from transcripts, and the names mentioned during the interview were replaced with pseudonyms to protect the identities of the participants. Each parent received $15.00 as compensation for their time.

The process of data analysis was guided by the principles of the constant comparison method [32], which allowed structural corroborations among members of the research team. The analysis steps included two cycles of coding as described by Saldaña [33]. For the purposes of the first coding cycle, each researcher was assigned to 1 of 2 analysis teams. The principal researcher and 2 other researchers participated in both teams. First, researchers thoroughly read the transcripts assigned to their respective team and individually identified initial categories. Then, individual researchers met with their respective teams to read transcripts as a group and discuss/agree upon emergent categories.

In the second coding cycle, portions of transcripts were organized into tables presenting mothers' and fathers' responses to the same questions side by side for intracouple comparisons of responses. Then, couples' responses were examined side by side for intercouple comparisons. During this cycle, researchers met several times to revise codes, share analytic memos, and reconfigure codes from the first cycle by linking interview chunks and/or initial codes. A total of four major patterns/themes were identified from the data: (1) general context of the playing experience; (2) playfulness in the context of parent-child interactions; (3) self-efficacy during play; and (4) motivations vs. benefits.

In qualitative studies, rigor is determined by the criteria of credibility, dependability, confirmability, and transferability [26]. Broad descriptions of the data collection and analysis process serve to reinforce credibility and dependability. Confirmability is achieved by showing that results are derived from a well-documented research process and through informants (e.g., participants' direct quotes) that support the results [34]. Transferability of the findings is demonstrated when practical implications derived from the findings are pertinent to other contexts [34].

## 3. Results

The following results include verbatim excerpts of participant narratives selected to exemplify the underlying themes. Identifying information has been removed from quotations. The verbatim texts were translated into English by the Translation Center, College of Humanities (University of PR), for the sole purpose of this article. Preservation of essential meaning, content, and, insofar as possible, general tone were the main goals of the translation. To enhance clarity and preserve the parents' anonymity, participants are identified with numbers 1 to 4 (e.g., mother number 1 and father number 1 form a parental couple). Each participant's number is shown at the beginning of each quotation.

### 3.1. General Context of the Play Experience

Prior to in-depth examination of the excerpts as they relate to the research questions, it is pertinent to contextualize participants' perspectives about play experiences with their children. In general, parents' play experiences were defined by codes related to emotions regarding play, relevance of play as part of a routine, and awareness of the child's diagnosis. All participants expressed positive emotions related to play experiences with their children. Satisfaction, happiness, and trust were among the most frequently expressed emotions. Participants also commented about the importance of playing with their children; their narratives reflected that moments for playing are specified as parts of the family routine. Generally, fathers indicated that they played with their children after arriving home from work. In contrast, mothers more frequently commented about how they integrate play into household chores and childcare activities:

Mother 1: *We are playing from the moment we wake up, from the moment we are brushing our teeth we are already playing because it's a brushing teeth game, it's another one to get dressed, and that's how we move forward …*

Father 1: *(We play) When I get home from work, before going to sleep… Like almost all of the time I am home, I try to spend it with my daughters and we are always playing*

Mother 3: *Look, I can be cooking, and he comes to the kitchen because he helps me a lot… He's very collaborative and he comes to the kitchen and wants to add sauce very carefully, right [… And while …] the food is getting ready, we have a tickle or kiss game…*

Mother 4: *[…] When we get home, dad and I get turns. Dad makes dinner and mom helps with homework. I start playing with him while dinner is ready. After dinner, I make the next day's lunch and dad plays with him, that is to say, we switch*.

Narratives about the difficulty of incorporating play encounters into ordinary routines were also very common among the participants. Main reasons for this challenge were related to the time demands of complex daily routines as well as to the inherent social-emotional challenges faced by children with ASD:

Mother 1: *Sometimes it's complicated because she's not always in the mood to play or learn or to do things or every day activities such as getting dressed or whatever. But, that's why it's simpler with play, because there are moments in which [we lose control and] we start yelling and we see how agitated she is and truth is nothing will be solved that way. We have learned… we'll take it slowly, we'll take a breath, and we'll do it a different way, and playing, well, makes it simpler…*

Mother 4: *Well, we are not at home most of the day; we work from 8 (am) to 7 at night. So, he is in activities most of the day, but when we get home, dad and I take turns [to play]*.

Father 4: *Obligations… like you have to do the laundry, you have to cook … Here we both do the chores; my wife does some and I do some others. Sometimes my wife may be there (points to the dining room) working and I am over here making dinner for him and sometimes that moment, well, I am not dedicating it to him… Well, I do dedicate it to him because it's for him, but I am not playing with him. You get me?*

Mother 3: *Well look, in some aspect it is (easy for the boy to play), what happens is that there are moments where he wants to play and […] I am a mom and I do not stop working and I still have responsibilities and, the truth is that, as a person, I get exhausted …*

Participant narratives also denoted awareness of the challenges associated with their child's diagnosis. In the case of Mother 2, it was possible to perceive some frustration about the ways in which she was able or unable to play with her daughter:

Mother 2: *[…] When I notice we start playing (while moving a toy she's holding and acting like paying) “hi, how are you?” she hates that… when I grab a toy and [try imaginary play], she hates it. And the anxiety of not being able to accomplish it… It freezes me […]. Because I get so anxious that “I must do it, and I have to do it” and that [anxiety] I need to control. It's something that “kills” me (laughs)*.

Another way in which participants denoted consciousness about the child's diagnosis was by highlighting the importance of play interactions as a mean to promote and follow their child's progress:

Father 1: *Well, the moments I enjoy the most, sometimes are when, when she does something that surprises me. In other words, let us say, well, that she does not pay attention to some things or she's very reluctant to do so and so activity and in that moment, you are doing something, and she comes and starts doing what you are doing and we are getting along. (…) It's said that children [on the spectrum], do not let people hug them, that [they avoid] visual contact, that they do not let… And you can hug that girl! You can be with her, she comes to me when I get home, she jumps at me! I enjoy those things because I thought they would not happen*.

Mother 2: *Yes, (play has its benefits) because I can tell you that six months ago, we could not [play]. Then, as a mom and human being who has been there, I was very frustrated, right, over the diagnosis … [But play] helps me to feel that we are moving forward, that it's worth the effort and it makes me feel that we are doing it right because we are seeing progress*.

### 3.2. Playfulness in the Context of Parent-Child Interactions

Analysis of participant narratives revealed the elements of playfulness embedded in parents' experiences. It was clear that play was often shaped by the child's preferences, indicating that parents sought interactions that were *intrinsically motivating* for their child. Most parents explained that the best way to play with their child was by immersing themselves in the type of play the child had already established, thus reflecting the element of motivation proposed in Skard and Bundy's model [6]. From parents' perspectives, parent-child interactions were more effective and enjoyable when play was intrinsically motivated rather than when the child's play preference was disrupted or when parents tried to impose their own choices on children during the play interaction:

Mother 2: *I try to play by her rules, and to not impose on her my way of playing. I can deal with her a little better when we play by her rules*.

Father 1: *… if you want to force the game she'll get difficult, but difficult as in she'll want to hit you and everything because she's doing something and you cannot interrupt what she's doing; because she'll start kicking at you and [yell] “I don't want to” and there will not be any break*.

Mother 4: *I try to play the same games he likes to encourage him*.

Father 2: *It's easy [to play]… The only thing I have to do is show her something she likes, and it's over. I show her the water bomb, and that's it, we start playing*.

*Internal control* was another element apparent in the narratives. Parents indicated that parent-child interactions were mainly arbitrated by the child, meaning that the child maintained overall control within play encounters. Although parents had some influence over some activities, they were mostly regulated by the child's preferences and feedback during the parent-child interaction. Parents indicated that children enjoyed play encounters and that interactions were reciprocal and occurred for longer periods of time when the child was in control. Both, mothers and fathers, denoted that when play preferences were forced, children tended to avoid play by displaying tantrums or trying to change play activities. This corresponds to Skard and Bundy's model [6]. When children feel in control and feel no constraints during play encounters, the outcomes of interactions are more effective, especially during parent-child interactions.

Father 4: *… it's easy [to play] as long as he likes the kind of play, it's easy. It's going to be very difficult if it's something he does not like…*

Father 2: *The hardest part, well, when the play is guided, you know there is free play and guided play […] if it's a guided game, well, it is harder because setting a pattern with a structure is not so easy with her, but if it's a free activity you can spend the whole day playing*.

The element of *framing* was also noted in the narratives. Often, mothers and fathers indicated a desire to prolong play encounters. When the child's preference or interest during an activity was interrupted, parents resorted to framing or cueing the child to continue the play process. Main framing strategies included the presentation of preferred toys or games that the child would enjoy and the initiation of cues (or responses to child cues) for playing. These strategies prolonged attention and participation during play encounters.

Father 1: *I get one of her favorite toys or I take her blanket away from her to make her come after me because she loves blankets and she takes with her 4 or 6 blankets wherever she goes […] Well, since I know she likes them, I take it away from her and I run to the bedroom and she comes looking for it. So, that's the moment I take to start playing. It's the same as with the little dolls [or other toys she love]… those are the little tricks I use [to play with her] (laughs).”*

Father 2: *She does not give up. When I get home, she tells me, “Seated” and then grabs me, pulls me by my arm, and takes me to the room and sits me on the floor (smiles) [saying:]. “Seated, let's play” and from there we start playing with anything that's available*.

Mother 2: *We are always having to create playtime in a way that gets her attention, whether cheering: “Yeah, Betsy, that's it, very good, we're going to play!” (lifting her arms in a cheering motion)… Very energetic, so it like grabs her attention and then she comes. That's what gets her*.

Furthermore, parents' descriptions of play encounters with their children reflected the element of *freedom to suspend reality*. It was relatively easy to observe simple forms of reality suspension such as teasing or rule stretching, especially among fathers. For example, Father 1 talked about play-wrestling with his daughter *(“She knows the mood I have. She knows my attitude […] Sometimes, she even knows what I want to do. You know if I pick her up and take her to bed, she knows that we're going to play wrestle or something like that… Then she gets excited, she gets motivated.”).* This brief excerpt from Father 1 denotes, not only the suspension of reality by stretching the rules (fathers do not typically wrestle with their daughters) but also the importance of his playful attitude (“*She knows the mood I have. She knows my attitude*…”) to cue the daughter and frame the play experience. Another strategy for reality suspension was pretending to be an object (e.g., a doll or action figure); however, this was not effective for all participants. While Father 1 indicated the use of little dolls to maintain his daughter's attention during play, Mother 2 confessed that she would love to engage in more imaginary play with her daughter and suggested that this affected her sense of self-efficacy during play activities:

Mother 2 *… I would like to make her playtime last longer. That's why, I do not feel a hundred percent satisfied, even though I feel happy that I am able to play with her… but it's really frustrating because you have to pull off this cooperative game and… I am like, “Here goes the car: beep beep.” (Moving her hands like she's driving a car), but she does not like that. She does not like it to the point that she will even get rid of the toys, and take them away from me… She'll play with me; but it has to be dolling me up, putting makeup on me, doing my nails, but anything that has to do with grabbing toys or having conversations, she does not like it. That's where I am frustrated, I would love to be able to do that a little*.

### 3.3. Self-Efficacy during Play: Motivations vs. Benefits

All participants communicated feelings of efficacy and satisfaction regarding play experiences with their children. Perceived ability to interact with their child was the main aspect that made parents feel competent. Playing provided parents with an opportunity to relate to their children and strengthen parent-child bonds. Additionally, parents connected play efficacy with positive feelings experienced during play moments, such as the emotion of seeing a child participating in, completing, and enjoying a play activity:

Father 1: *I mean, I consider myself pretty effective. Any person can come along, even her mother, and tries to do what I do, and it will not work … I mean, I have my way, my tricks… because it's a matter of motivating her to play, to take her the things she likes. If she likes dolls, or if you see on this day she woke up wanting her blanket or on that day she woke up wanting dolls… you already know which way to go to motivate her to play with you*.

Mother 1: …*For example, we were playing um … bowling, and then, it happened that she knocked down the pins, and you see her joy… it's like “I did it!”, like she feels successful*.

Overall, fathers verbalized feelings of better efficacy during play experiences with their children than did mothers. This observation is exemplified by the excerpt from Father 1 shown above. Indeed, mothers were more likely to identify aspects of the play process that they wanted to improve or change:

Mother 1: *[I would like to get better] as far more improvisation […] and… I do not know maybe a little more athleticism, running harder*.

Mother 2: *I still need to learn a little more, how to get through to her, how to get her a little more interested, uh, work a little more in that area*.

Mother 3: *[How effective I feel] depends really on the game. There are games where like … for example PlayStation: [He invites me] “Mommy come play with me.” Now, there are other games, for example, painting… Surprisingly, that does not really get his attention. So, since I have to struggle a little bit for him to sit with me […] it's a little challenging to get him interested and it's like (sighs) let me see what else I can do to get his attention*.

The analysis also identified different *motivations for playing* between fathers and mothers. It was notable that fathers' spontaneous responses about motivations for playing reflected a greater emphasis on emotional aspects such as seeing their children laughing, smiling, and happy, while mothers were motivated to play to promote progress and enhance their child's necessary skills. This difference is shown in the excerpts below, presented by a parental couple (e.g., Mother 1 followed by Father 1):

Mother 1: *[It motivates me] to see that you can accomplish a lot of things through playtime [for] example, writing, reading, colors, everything… Playing … is the easiest way of teaching these children*.

Father 1: *Seeing her laugh, seeing that she's having a good time; also, we can learn too…*

Mother 4: …*Helping him, more than anything, to socialize, that when he sees the neighbors or his friends outside, he can be sociable […] and that he spends time with them, that he does not become a shy person and above all, share with people*.

Father 4: *The first thing is the love I have towards him, as my son… that motivates me*.

Interestingly, when asked about the perceived *benefits* of playing experiences, an opposite trend was observed. Mothers' spontaneous responses tended to emphasize more emotional benefits of play (e.g., trust and bonding) while fathers were likely to identify aspects related to the child's progress or skill development.

Mother 3: *Aside from working on what we call quality time for both […] Playing develops trust. He feels how we speak during playtime. So, he gets aware that: “Oh, I can speak with mommy”, or “I can tell things to mommy”, and since he's young now, it's important to develop this confidence and this communication with him… Playtime helps a lot with this. In addition, we have fun and we laugh together. So, when he gets older he's going to say: “I remember when mommy played that with me…”*

Father 3: *[…] Oh, yes, it [play]soothes him, it helps him to go to bed more relaxed*.

Mother 1: *Yes [play is beneficial]. As it pertains to confidence, I think my daughter pretty much trusts me …*

Father 1: *Yes, because [in addition to strengthens the attachment], she develops a lot better, she accepts physical contact. If I had never played with her, if I had never become interested in playing with her, well, she would not want contact with anybody […Also,] I think movement [coordination] is developed, I mean, before, she would trip over herself a lot. Sometimes, she did not even want to play. But now, not anymore. Now she's well-coordinated, very agile, and she has great equilibrium!*

## 4. Discussion

The purpose of this phenomenological study was to explore the play experiences of parents with children on the autism spectrum. First, we focused on parents' narratives reflecting playfulness while playing with their child. Consistent with previous research, there were characteristic differences in the contexts and types of play used by mothers and fathers [35–37]. Mothers tended to insert play moments into daily routines while fathers had opportunities for playing that were not necessarily embedded within other daily tasks.

We were able to identify elements of Skard and Bundy's model of playfulness [6] in participant comments. Mothers and fathers assigned importance to facilitating their child's *intrinsic motivation* and *internal control* and recognized that, by doing so, they were better able to engage the child in play. Skard and Bundy [6] stated that internal control requires that all participants in the play experience can make decisions regarding the details of the play encounter, thus implying a certain degree of negotiation. Yet we found that when parents, specifically fathers, wished to see the child's enjoyment during play moments, they opted to relinquish internal control to a substantial extent.

Parents' comments indicative of *framing* were generally efforts to offer “play cues” to children. The message “this is play” was deliberately linked to things that intrinsically motivated the child. The observed relationship between intrinsic motivation and framing is not surprising. Rather, we were interested in the “tangible quality” of the framing cues used by parents (or the child; e.g., the use of specific objects or a very energetic attitude). Although these types of cues can be used by any parent with any child, it is possible that tangible qualities have particular importance in families with children on the autism spectrum. Framing and scaffolding, although not equivalent, are conceptually related to one another. Various terms that refer to scaffolding strategies (e.g., prompting, modeling) can also be used as cues to portray a play situation to the child [38–40]. Social participation difficulties are thought to interfere with the development of complex play skills in children with ASD and might make it more difficult for parents to scaffold play [39]. Further, evidence suggests that children with ASD rely on others to generate novel ideas about how to play to a greater degree than do typically developing children [41].

Pretend play is the most common form of *suspension of reality* [7]. Children with ASD have a reduced preference for this type of play [42–44]. Experiences of participants in this study corroborated this idea and generally recounted less obvious suspensions of reality such as teasing (e.g., the father running with his daughter's blanket) or stretching the rules (e.g., the father play-wrestling with his daughter), especially in the narratives of fathers. We also recognized challenges related to engaging the child in pretend play situations. One mother verbalized significant frustration about not being able to actively engage her daughter in pretend play. This is consistent with previous evidence; in a cross-sectional study, Case-Smith and Kuhaneck [45] found that children with developmental delays tended to prefer rough-and-tumble play and object exploration and presented lower preferences for drawing and coloring, construction, and doll and action figurine play.

Our results indicated that, in terms of playfulness, facilitating the elements of framing and suspension of reality was more challenging than facilitating child's intrinsic motivation and internal control. This is in line with previous research stating that the number of imitated actions and amount of elaborate pretend play (both areas of difficulty for children with ASD) are positively associated with the suspension of reality and framing dimensions of playfulness, respectively [41]. Despite the difficulty of certain elements, it was perceived that participants assumed an important role in facilitating playful experiences with their children and that the playful attitude of parents was an important facilitator or barrier in this context.

The second question guiding our analysis in this study was how competent do fathers and mothers feel when playing with their child with ASD? Perceived ability to interact with the child and positive emotions experienced during play moments were among the main aspects that participants associated with self-efficacy during play. Fathers derived a greater sense of self-efficacy from play encounters while mothers were more likely to identify areas of their own performance that they wanted to improve. A key factor explaining differences in the perceived efficacy of mothers and fathers during play is their perceptions about the motivations for playing and the benefits associated with the play experience.

As stated, an ability to interact with children and the derivation of positive emotions from play interactions were identified by all participants as indicators of efficacy. The analysis of fathers' narratives reflected a better match between motivators for playing and the aforementioned indicators of efficacy. While fathers' motivations depicted a greater emotional emphasis (e.g., *“seeing her smiling”*), mothers' motivators were more outcome-oriented (i.e., more focused on promoting the child's development of skills; e.g., *“help him to socialize better”*). Interestingly, the opposite was observed when analyzing the benefits that parents derived from play moments. Fathers identified benefits that were more outcome- or task-oriented (e.g., ability to move more efficiently; a more relaxed state at sleeping time) while mothers identified more emotionally oriented benefits (e.g., own enjoyment and enhancement of the child's trust in them). Considering that fathers were more inclined to judge themselves as competent during play than mothers, the findings of this study suggest that a better match between motivators for playing and perceived indicators of efficacy during play are relevant to a parental sense of self-efficacy.

The present findings have several important implications. First, it may be useful to help parents to better understand the difference between play or playfulness and the elements of playfulness. A comprehensive understanding of the elements of playfulness can guide parents in equalizing the importance attributed to play as a means as well as an end. Too often, parents (in the case of this study, especially mothers) of children with ASD and other disabilities are so overwhelmed by delays in their child's development that they forget the importance of *playing playfully* and making play encounters a pleasurable experience for everyone involved. The enjoyment of these encounters not only addresses the child's challenges in social participation but also enhances parental self-efficacy [19].

Participants in this study cared about having opportunities to relate to and play with their children, but some (mothers more than fathers) were unable to derive a sensation of self-efficacy from playing. Education-based treatments may help parents like these to adjust their perceptions to improve consistency between definitions of efficacy during play and motivations to play, resulting in the enhanced perception of parental self-efficacy. Occupational performance coaching (OPC) can be an alternative. OPC is an educational treatment that uses collaborative problem-solving within a coaching relationship in which parents are guided to identify and apply effective solutions to occupational performance problems with their children [46].

This is particularly significant in the context of occupational therapists' family-centered interventions, since role competence is an important desired intervention outcome [47]. Occupational therapists have the opportunity to use coaching, especially when working with families with children in early childhood [48, 49]. In terms of play, important reflections include the following: How are we focusing coaching processes? Are we balancing teaching families about play as a means and as an end? Evidence indicates that there is still a long way to go; a study by Kuhaneck et al. [50] found that only 4% of 198 pediatric occupational therapists reported the use of play as an intervention outcome.

On the other hand, differences between parents in motivators for playing might be attributed to parents' perceptions about what is expected from them based on traditional gender roles [51]. Research suggests that gender roles are critical for defining the implicit objectives of parent-child interactions [52]. Further, evidence suggests that playfulness is a trait with likely ties to environmental and interactional characteristics [53]. Thus, it is pertinent to help parents to visualize playfulness not only simply as a personality trait but also as an environmental and interactional component. Parents themselves are a key part of the child's environment. Additionally, traditional gender roles are, to some extent, part of the parents' environment and can influence parents' expectancies about play encounters with their children. This concept merits further exploration in future studies. In summary, the results of this study suggest that parents' expectancies and attitudes affect not only the way in which they approach play encounters but also the way in which their children approach the play experience; this in turn has effects on parents' sense of self-efficacy during play.

### 4.1. Limitations

This study contributes to a body of knowledge about play and playfulness in families of children with ASD and the potential relevance of these constructs for parental self-efficacy. The profiles of couples that participated in this study were relatively diverse. This allowed the collection of information covering a broad spectrum of family contexts (e.g., parents living together with more than one child; parents living together with only one daughter with ASD; parents with one son with ASD not living together; and parents living together with very demanding workloads). While this can be considered a strength, it must be recognized that this was a phenomenological study with a limited number of participants (4 parental couples and therefore 8 participants). We understand that having a limited quantity of participant couples along with such diverse profiles entailed the main limitation of this study, because it impeded reaching saturation. For example, the narratives of Mother 2 (who had an only daughter) depicted substantial frustration regarding play interactions with her daughter with ASD (e.g., *when I grab a toy and [try imaginary play]… she hates it. And the anxiety of not being able to accomplish it… It freezes me […]. Because I get so anxious…)*. On the opposite, Mother 1 (who had two additional typically developing daughters) did not describe comparable frustrations with her daughter with ASD. Having additional participant mothers with similar family context (e.g., more mothers with an only daughter with ASD and mothers with additional neurotypical daughters) would have allowed us to verify if their experiences and perceptions were similar.

The abovementioned issues give rise to areas deserving additional exploration in future studies, such as play styles of parents of children with ASD in the context of families with multiple children, between dyads of mothers/fathers-daughters with ASD, among mothers/fathers-sons with ASD, and in the context of families in which parents do not live together or in which children are under the care of family members other than the parents. Additionally, it would be worthwhile to evaluate our findings against the narratives of parents with neurotypical children to verify whether the ability to sustain and enjoy play encounters with their children is linked in any way to their sense of self-efficacy. Finally, we recommended that future studies examining areas explored in this study utilize more comprehensive qualitative designs such as grounded theory.

## 5. Conclusion

The purpose of this study was to explore the perspectives of parents of children on the autism spectrum regarding play experiences and self-efficacy during play encounters with their children. Specific attention was given to elements of playfulness and parents' sense of self-efficacy during play encounters with their children. Participants' narratives indicated that facilitating framing and suspension of reality was more challenging than facilitating the child's intrinsic motivation and internal control during play. Finally, the results suggest that a better match between motivators for playing and perceived indicators of efficacy during play has a significant relevance for parental self-efficacy. Occupational therapists should employ coaching strategies to help parents increase their understanding of play and playfulness and how these elements can affect their sense of self-efficacy.

## Figures and Tables

**Table 1:** Description of participants.

Participants	Age	Gender and age of the child with ASD	Couple living together/apart	Additional members of the family
Mother 1	25	Female, 7	Together	2 sisters (5 and 3 y/o, respectively)
Father 1	31			

Mother 2	38	Female, 4	Together	No additional members
Father 2	43			

Mother 3	27	Male, 5	Apart	Child lives with mother but stays with father frequently. Has a brother (14 y/o) on his father's side, who lives with the father
Father 3	43			

Mother 4	40	Male, 5	Together	No additional members
Father 4	45			

## Data Availability

This was a qualitative study. Data consists of participant quotes extracted from ad verbatim transcripts of semistructure interviews.
